# Assessment of Activity of Daily Life in Mucopolysaccharidosis Type II Patients with Hematopoietic Stem Cell Transplantation

**DOI:** 10.3390/diagnostics10010046

**Published:** 2020-01-16

**Authors:** Yasuyuki Suzuki, Madeleine Taylor, Kenji Orii, Toshiyuki Fukao, Tadao Orii, Shunji Tomatsu

**Affiliations:** 1 Medical Education Development Center, Gifu University, Yanagido 1-1, Gifu 501-1194, Japan; 2 Nemours/Alfred I. Dupont Hospital for Children, 1600 Rockland Rd., Wilmington, DE 19803, USA; taylormm@udel.edu; 3 Department of Pediatrics, Gifu University Graduate School of Medicine, Yanagido 1-1, Gifu 501-1194, Japan; kenjior-gif@umin.ac.jp (K.O.); toshi-gif@umin.net (T.F.); tomatsushunji@gmail.com (T.O.); 4 Department of Pediatrics, Thomas Jefferson University, Philadelphia, PA 19107, USA

**Keywords:** mucopolysaccharidoses, hunter syndrome, activity of daily living, hematopoietic stem cell transplantation

## Abstract

The effectiveness of hematopoietic stem cell transplantation (HSCT) for mucopolysaccharidosis type II (MPS II, Hunter disease) remains controversial although recent studies have shown HSCT provides more clinical impact. This study aims to evaluate the long-term effectiveness of HSCT using the activity of daily living (ADL) scores in patients with MPS II. Sixty-nine severely affected MPS II patients (19 patients who received HSCT and 50 untreated patients) and 40 attenuated affected patients (five with HSCT and 35 untreated) were investigated by a simplified ADL questionnaire. The frequency of clinical findings and the scores of ADL (verbal, gross motor, and the level of care) were analyzed statistically. The mean age of onset of 19 severely affected patients who received HSCT was 1.40 years ± 1.06, which is not statistically different from that of 50 untreated patients (*p* = 0.11). Macroglossia, frequent airway infection, hepatosplenomegaly, joint contracture, and sleep apnea were less frequent in the HSCT-treated group of severe MPS II patients. The severe phenotype HSCT treated group reported a statistically significant higher score of verbal function and gross motor function between the ages of 10 and 15 years and a higher level of care score between 10 and 20 years. Patients with the attenuated phenotype showed high ADL scores, and all of five HSCT treated patients reported a lower frequency of frequent airway infection, coarse skin, umbilical/inguinal hernia, hepatosplenomegaly, heart valve disorders, and carpal tunnel. In conclusion, HSCT is effective, resulting in improvements in clinical features and ADL in patients with MPS II. HSCT should be re-reviewed as a therapeutic option for MPS II patients.

## 1. Introduction

Mucopolysaccharidosis (MPS II, Hunter disease, iduronate-2-sulfatase deficiency, OMIM #309900) is a lysosomal storage disorder with X-linked recessive inheritance characterized by the systemic manifestations such as short stature, multiple joint contractures, dysmorphic face, airway narrowing and infection, heart valve disorders, hepatosplenomegaly, visual and hearing difficulties and neurologic disabilities [1,2]. Accumulation of glycosaminoglycans (GAGs) such as dermatan sulfate (DS) and heparan sulfate (HS) in the body causes these progressive manifestations. Patients with the severe phenotype show cognitive impairment and neurological deterioration that lead to the early death before adulthood, whereas patients with the attenuated phenotype usually manifest progressive cardiopulmonary insufficiency, hearing impairment, and gradual decrease of the activity of daily living (ADL) in the adulthood. Hematopoietic stem cell transplantation (HSCT) had been widely performed as a therapeutic approach for patients with MPS I (Hurler syndrome) since the 1980s [3,4]. However, HSCT for patients with MPS II has been controversial, and the early studies suggest that HSCT is not effective for MPS II [5,6]. Since enzyme replacement therapy (ERT) for MPS II has been introduced into practice, clinical benefits of ERT including physical endurance, pulmonary function, range of joint motion, hepatosplenomegaly, coarse hair and skin, as well as the reduction of urinary excretion of GAGs have been reported [7,8]. Brain disorders, bone dysplasia, and valvular heart diseases remain an unmet challenge by the current ERT approach. In a more recent study, Kubaski et al. [9]. In 2017 reported the effects of HSCT on 146 MPS II patients. The overall conclusion was that HSCT improved ADL, the findings by brain magnetic resonance imaging (MRI), and GAGs levels. Other reports on MPS II patients with HSCT have supported a positive impact on the ADL and growth especially if HSCT is performed at an early stage [10,11,12,13,14,15,16]. While HSCT provides considerable risks such as graft-versus-host diseases, infections, and higher mortality rate during the treatment, it also has unique advantages compared with ERT: lower total costs, one-time treatment if successful, and the potential CNS effects if treated at an early age [9,13,17,18,19,20]. In this study, we have investigated the effectiveness of HSCT for patients with MPS II, using a simplified ADL questionnaire in Japan. Our finding further demonstrates that HSCT improved clinical manifestations and ADL of the patients with the severe phenotype.

## 2. Materials and Methods

The investigators at Gifu University sent the first questionnaire to 8969 departments (Pediatrics, Internal Medicine, Orthopedics, Rehabilitation, Ophthalmology) of hospitals with 200 beds or more and asked whether these institutes had patients with MPS II. The recovery rate of the first questionnaire was 27.0%. One hundred forty-six patients with MPS II (99 patients with the severe phenotype, 47 patients with the attenuated phenotype) had been reported to Gifu University. The second questionnaire was sent to each institute who had MPS II patients(s). This questionnaire included demographic data, clinical manifestations, therapeutic management, and ADL in these patients investigated further. Data on 109 of 146 (74.7%) patients with MPS II (69 patients with the severe phenotype and 40 patients with the attenuated phenotype) were collected (Table 1) between the years of 2000 and 2007. All subjects were Japanese. The questionnaires were filled out by the patient and family, based on the patient’s current symptoms. ERT for MPS II patients was approved in Japan in 2006 [21]. Two untreated patients with the attenuated phenotype reported receiving ERT. This does not impact our conclusion for HSCT. The simplified ADL scoring table was developed as shown in Table 2. This table is a simplified version of the validated FIM that has been used to evaluate ADL scores [22]. Verbal function, gross motor function, and the level of care in each patient were evaluated based on this scoring system and defined in Table 2. Rates and proportions were compared using the *t*-test or χ^2^ test. This study was approved by the Institutional Review Board of Gifu University (approval code: 23-233, approval date: 1 January 2012).

## 3. Results

### 3.1. Patients’ Characteristics and Clinical Manifestations

Demographics of 109 patients with MPS II are summarized in Table 1. Sixty-nine and 40 patients were categorized into the severe and the attenuated phenotypes based on the clinical severity and the presence of cognitive impairment. Mean age at onset, age at diagnosis, and the age at this study in the patients with the severe phenotype were significantly younger (*p* < 0.01) than those with the attenuated phenotype. Nineteen out of 69 (27.5%) patients with the severe phenotype received HSCT. Statistical differences between the age at onset, diagnosis, and at time of the study were not observed between HSCT-treated and untreated groups for both the severe and the attenuated phenotypes. Only 5 out of 40 (12.5%) patients with the attenuated phenotype received HSCT.

In the untreated patients with either of the severe or attenuated phenotype, facial dysmorphism, frequent airway infection, coarse skin/hypertrichosis, umbilical/inguinal hernia, hepatosplenomegaly, joint contracture, heart valve disorders, and hearing difficulty were common clinical features. Macroglossia, sleep apnea, and cognitive impairment were more frequently observed in patients with the severe phenotype. Carpal tunnel syndrome and corneal clouding were observed in a smaller percentage of the patients. In the HSCT-treated group with the severe phenotype, macroglossia, frequent airway infection, hepatosplenomegaly, joint contracture, and sleep apnea were less frequently observed when compared with the untreated patients. In the HSCT-treated group of the attenuated phenotype, airway infection and coarse skin/hypertrichosis, umbilical/inguinal hernia, hepatosplenomegaly, joint contracture, heart valve disorders, and carpal tunnel syndrome were less frequently observed.

### 3.2. Natural History of ADL of MPS II

Data from the questionnaires were used to score the ADL of patients based on a simplified ALD scoring table (Table 2).

**Figure 1:**
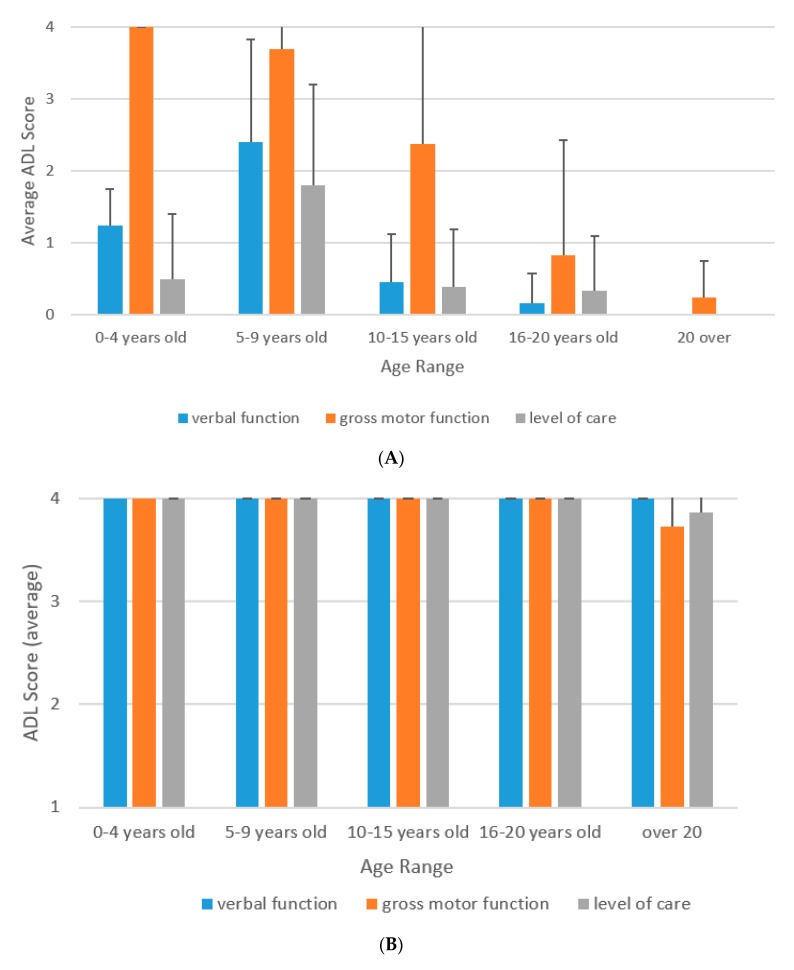
(**A**) ADL scores for untreated severe MPS II patients. (**B**) ADL scores of untreated attenuated MPS II patients.

Scores of ADL in untreated patients with severe or attenuated phenotypes are graphed in Figure 1. Data from the untreated patients provide a natural course of ADL in MPS II patients. In untreated patients with the severe phenotype, the mean ADL score of verbal function between the ages of five and nine years was slightly higher than that of ages four and below, but it decreased rapidly at 10 years of age (Figure 1A). The mean score of gross motor function was highest (ordinary walk) at four years of age or younger, but gradually decreased as age progressed (Figure 1A). The mean score indicated that the patients with the severe phenotype could walk with aid between five and 15 years; however, they became wheelchair-bound after 15 years of age, and then bed-ridden in adulthood (Figure 1A). The level of care was highest between the ages of five and nine and decreased after nine years of age (Figure 1A).

In the patients with the attenuated phenotype, verbal function, gross motor function, and the level of care were maintained in normal up to 20 years of age; however, the score of gross motor function and the level of care slightly declined after 20 years of age (Figure 1B).

### 3.3. ADL of Patients Treated with HSCT

**Figure 2:**
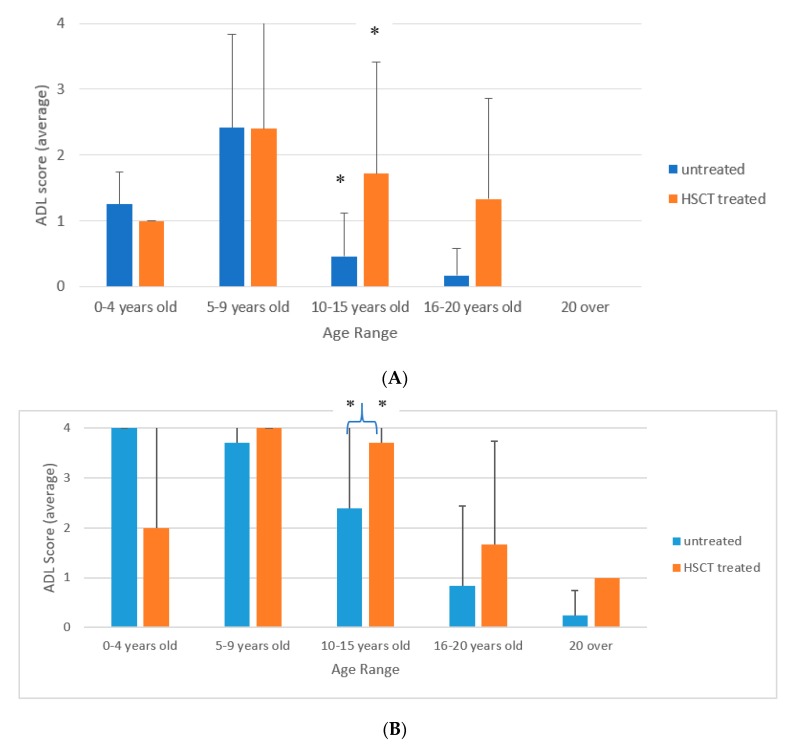

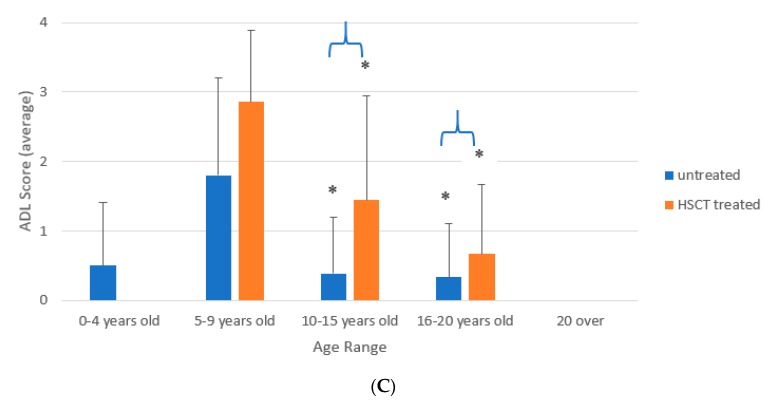
(**A**) Verbal function ADL scores of severe phenotype HSCT-treated and untreated groups. * = *p* < 0.05. (**B**) Gross motor function ADL scores of severe phenotype HSCT-treated and untreated groups. * = *p* < 0.05. (**C**) Level of Care ADL scores of severe phenotype HSCT-treated and untreated groups. * = *p* < 0.05.

**Figure 3:**
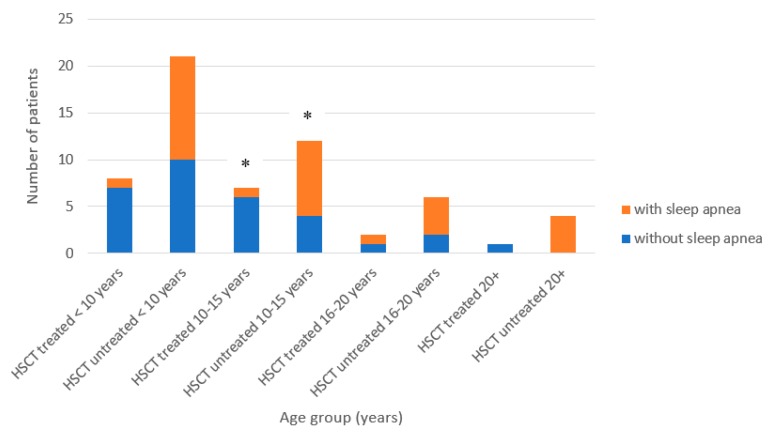
Sleep apnea in severe phenotype MPS-II patients. * = *p* < 0.05.

ADL scores were compared between HSCT-treated vs. untreated patients with a severe phenotype (Figure 2). There were no statistical differences in the ages at the onset, the diagnosis, and age at study between the HSCT-treated vs. untreated severe MPS II patients. As described above, macroglossia, frequent airway infection, coarse skin, hepatosplenomegaly, joint contracture, and sleep apnea were less frequent in patients who received HSCT (Table 1). Sleep apnea was significantly less often observed in the HSCT-treated patients for the ages between 10 and 15 years (Figure 3).

**Figure 4:**
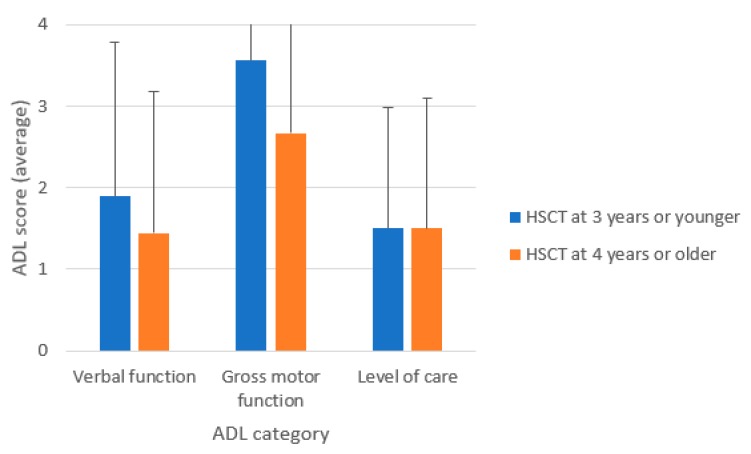
Age at HSCT and ADL scores for severe phenotype MPS II patients.

As shown in Figure 2, ADL scores of HSCT-treated patients were generally better than those untreated patients. The score of verbal function was not different between both groups before 10 years of age but was significantly higher in the HSCT-treated group between 10 and 15 years of age (Figure 2A). The score of gross motor function remained relatively high between five and 15 years of age. The ADL scores of the HSCT treated group was significantly higher in the HSCT-treated group compared to the untreated group between 10 and 15 years of age (Figure 2B). The level of care was significantly better in the HSCT-treated group between 10 and 15 years old and between 16 and 20 years old. However, both groups became highly age-dependent after 15 years of age (Figure 2C). ADL scores in HSCT-treated patients at three years of age or younger (*n* = 9) were slightly better than that of patients who were HSCT-treated at four years of age or older (*n* = 9) (Figure 4) (*p*-value: verbal 0.28, motor 0.11, care 0.5).

Note; Results of ADL survey for MPS II patients for the categories verbal function, gross motor function, and level of care. Rates and proportions were compared using the *t*-test or χ^2^ test.

## 4. Discussion

In this study, we have demonstrated a better clinical outcome and ADL in a relatively large number of HSCT-treated patients with the severe phenotype. Clinical effectiveness of HSCT for patients with MPS II remains controversial [9,13,17,18]. Peters et al. indicated that all the patients with the severe phenotype who received HSCT reported deteriorated intellectual abilities after HSCT (North American Storage Disease Collaborative Study Group) [23]. He also raised a question of ethical concerns on the decision-making of HSCT for MPS II with the attenuated phenotype, because of life-threatening risks of HSCT. In contrast, other investigations suggested that selected patients without serious neurological complications could be candidates for HSCT [24,25]. Guffon et al. reported that HSCT was effective on eight MPS II patients with a broad clinical range and non-neurological symptoms [26]. Scarpa et al. [27] reviewed that HSCT could provide sufficient enzyme activity to slow or stop the progression of the disease. However, controlled clinical studies should be conducted. A study performed by Kubaski et al. [9] has demonstrated that HSCT improves ADL scores, MRI findings, cognitive function, and reduction of blood GAG levels in severe MPS II patients [9,10,11,12,13,14,15,16,19,28]. There was little improvement in HSCT treated attenuated MPS II patients. However, there were some improvements in the patients’ MRI findings, which could suggest that HSCT could delay neurological symptoms in attenuated phenotypes. With these findings, Kubaski et al. has suggested that HSCT is a good therapeutic option for MPS II, but notes that the earlier HSCT is performed, the better the outcomes. Our findings in this study support for the findings in these recent studies.

MPS II is the most prevalent type of MPS in Japan [29] and in East Asia [30,31,32,33], and a considerable number of patients with MPS II have received HSCT in China and Japan [10]. However, only a few systemic evaluations on the effectiveness of HSCT have been performed up to date [9,10,11,19]. In this study, we conducted a retrospective questionnaire-based study of MPS II patients in Japan and collected clinical data from 109 patients with MPS II. Differences in clinical findings and the level of ADL were defined between the patients with the severe phenotype and the attenuated phenotype, and between the HSCT-treated and untreated patients. Our analyzed data indicated better clinical consequences in symptoms of macroglossia, frequent airway infection, hepatosplenomegaly, joint contracture, and sleep apnea in severe phenotype HSCT-treated group. ADL scores in verbal function, gross motor function, and level of care are also better in the severe phenotype treated group. However, verbal function rapidly deteriorated from single-word level to no-word level after nine years of age in the untreated group, while such level is reasonably maintained in the HSCT-treated group. Gross motor function is preserved in both groups between five and nine years of age and between 10 and 15 years of age, but the untreated patients are wheelchair-bound or bed-ridden after 15 years of age, whereas the treated patients reported a slightly better ADL score average. The level of care is also significantly higher in the treated patients between 10 and 20 years of age.

These results indicate that HSCT for patients with MPS II is effective not only for the somatic symptoms but also for some neurological symptoms. HSCT has several advantages when compared with ERT. Transplanted stem cells could migrate into the central nervous system and differentiate to glial cells [34] and may have a more favorable effect on cognitive function compared to ERT [9,10]. Those receiving HSCT do not have to worry about an antibody response to the infused enzyme, which could occur in ERT patients [9]. Patients do not need a weekly 4–5 h infusion of the enzyme for the lifetime. The total costs of HSCT will be less expensive if the patient is fully engrafted by the one-time procedure. The one-time cost of HSCT in Japan ranges between USD 70,000 and 205,000 [3,9,17,18,19,20,35]. The annual cost of ERT for a 25 kg MPS II patient in Japan is USD 400,000 [9,13].

On the other hand, HSCT also has risks and concerns. Life-threatening graft-versus-host reactions and infections may occur in HSCT [36]. Although, recent studies have reported increased survival rates in MPS II, including a study by Wang et al. [10], which reported a 100% survival rate in a 10-year follow-up of HSCT treated MPS II patients. Long-term admission to a hospital and separation in a bio-clean room is stressful for both patients and families [37]. Furthermore, we have no definite evidence at present that HSCT should have sufficient efficacy for the severe patients MPS II although HSCT provides a better clinical outcome compared with conventional ERT [9,13,14,15]. Our present results indicate the better prognosis of HSCT-treated patients. However, the patients have still limited verbal and motor function as compared with the healthy age-matched children, and verbal and motor function are highly dependent on ages.

Further research of MPS II patients who are under two years of age at the time of HSCT, since a more clinical impact on the CNS of MPS I patients treated under two years old has been observed [9,11]. Investigation and establishment of strategies for the early diagnosis and early treatment for MPS II are needed. Newborn screening for MPS is essential to promote early diagnosis [38,39,40]. New delivery strategies of the enzymes into the central nervous system are also under developing [41,42,43,44,45,46,47,48,49,50,51,52,53].

In parallel with the development of therapeutic regimes like HSCT and ERT, a simple method for the evaluation of clinical severity and therapeutic efficacy is demanded. We previously reported the usefulness of the functional independence measure (FIM) questionnaire [22]. A clear difference in ADL and psychomotor development between MPS II patients with the severe and attenuated phenotype was defined. The FIM questionnaire can be used for various diseases with neurological involvement. However, it requires the evaluation by the experts and is time-consuming. In this study, we attempted to use a novel and simple scoring table to assess a critical sketch of ADL in an individual patient, leading to the success of the establishment of clinical severity score and clinical efficacy post-HSCT. This revised method can be used by physicians and caregivers repeatedly and easily, although it could be difficult to assess a subtle change of function in each patient.

In conclusion, we have evaluated clinical symptoms and ADL in patients with MPS II and have identified significant differences in symptoms and ADL between HSCT-treated and untreated patients with the severe phenotype of MPS II. These data should provide further evidence for not only the natural history of MPS II but also the efficacy of HSCT.

## 5. Limitations of the Study

Current scientific knowledge gaps in this study are: (i) lack of objectivity of the data resulting from the use of a self-reported questionnaire, (ii) lack of an objective evaluation method to cover the whole symptoms, and (iii) a limited number of patients with MPS II. Longitudinal and objective data should be included in future investigation.

## Figures and Tables

**Table 1:** Characteristics of patients with mucopolysaccharidosis (MPS II).

	Severe Phenotype		Attenuated Phenotype	
Number of patients (M/F)	69 (68/1)		40 (40/0)	
Age at onset (year)	1.68 ± 1.30 **		3.08 ± 2.28	
Age at diagnosis (year)	3.2 ± 1.80 **		7.08 ± 5.75	
Age at study (year)	10.85 ± 5.86 **		16.69 ± 8.02	
HSCT	+	−	+	−
Number of patients	19	50	5	35
Age at onset (year)	1.40 ± 1.06	1.87 ± 1.38	2.5 ± 2.18	3.14 ± 2.27
Age at diagnosis (year)	2.78 ± 1.77	3.36 ± 1.80	3.4 ± 2.07	7.64 ± 5.94
Age in this study (year)	10.89 ± 5.45	10.84 ± 6.07	13.0 ± 8.28	17.3 ± 7.95
Age at HSCT	4.11 ± 2.42		5.0 ± 2.74	
Clinical findings (%)				
Facial dysmorphism	89	100	100	100
Macroglossia	59 **	90	0 **	68
Frequent airway infection	50 **	83	20 *	76
Coarse skin/hypertrichosis	79	98	40 **	91
Umbilical/inguinal hernia	56	69	25 *	81
Hepatomegaly/splenomegaly	78 *	96	40 **	94
Joint contracture	84 *	100	60	100
Heart valve disorders	63	80	60 *	94
Corneal clouding	6	13	0	12
Hearing difficulty	67	85	60	77
Sleep apnea	11 **	63	0	35
Central nervous symptoms	89	98	40	47
Carpal tunnel syndrome	0	0	20 *	16
Dilated ventricle (MRI)	47	71	40	39

* *p* < 0.05 ** *p* < 0.01. Note; Mean and standard deviation reported for 109 of 146 patients with MPS II. Patients were surveyed for the phenotype of MPS II, hematopoietic stem cell transplantation (HSCT) treatment, and physical symptoms of MPS II.

**Table 2:** Scoring table for activity of daily living (ADL) in patients with MPS II.

Category	4	3	2	1	0
Verbal function	Ordinary conversation	Two-words sentence	Single-word	Babbling	No word
Gross motor function	Ordinary walk	Walk with stick	Walk with aid	Wheelchair	Bed-ridden
Level of care	Independent		Moderately dependent		Highly dependent

## Abbreviations

HSCT	Hematopoietic stem cell transplantation
MPS II	Mucopolysaccharidosis type II (Hunter disease)
ADL	Activity of living
GAGs DS HS ERT MRI FIM	Glycosaminoglycans Dermatan sulfate Heparan sulfate Enzyme replacement therapy Magnetic resonance therapy Functional independence measure
